# Plasma Complement C1q/tumor necrosis factor-related protein 15 concentration is associated with polycystic ovary syndrome

**DOI:** 10.1371/journal.pone.0263658

**Published:** 2022-06-14

**Authors:** Akram Vatannejad, Reza Fadaei, Fouzieh Salimi, Fatima Zahraa Fouani, Behnam Habibi, Somayeh Shapourizadeh, Samira Eivazi, Sadegh Eivazi, Asie Sadeghi, Nariman Moradi

**Affiliations:** 1 Department of Comparative Biosciences, Faculty of Veterinary Medicine, University of Tehran, Tehran, Iran; 2 Sleep Disorders Research Center, Kermanshah University of Medical Sciences, Kermanshah, Iran; 3 Department of Clinical Biochemistry, Faculty of Medicine, Kerman University of Medical Sciences, Kerman, Iran; 4 Department of Cellular and Molecular Nutrition, School of Nutritional Sciences and Dietetics, Tehran University of Medical Sciences, Tehran, Iran; 5 Department of Biology, Faculty of postgraduate, Borujerd Branch, Islamic Azad University, Borujerd, Iran; 6 School of Mohadeseh, Shahriyar Education Office, Ministry of Education, Tehran, Iran; 7 Department of Biology, Science and Research Branch, Islamic Azad University, Tehran, Iran; 8 Department of Anatomy, Faculty of Medicine, Iran University of Medical Sciences, Tehran, Iran; 9 Endocrinology and Metabolism Research Center, Institute of Basic and Clinical Physiology Sciences, Kerman University of Medical Sciences Kerman, Kerman, Iran; 10 Cellular and Molecular Research Center, Research Institute for Health Development, Kurdistan University of Medical Sciences, Sanandaj, Iran; Medical University of Vienna, AUSTRIA

## Abstract

Polycystic ovarian syndrome (PCOS) is a common poignant endocrine disorder affecting women, posing a close association with metabolic syndrome and obesity. Existing literature characterizes PCOS with deranged levels of several adipokines and myokines. CTRP15 is a paralogue of adiponectin, mainly expressed by skeletal muscles, and plays a key role in insulin, glucose, and lipid metabolism. In the current study, we aim to determine the circulating levels of CTRP15 and evaluate its association with cardiometabolic and inflammatory parameters in PCOS women. This case-control study included 120 PCOS patients (60 Recurrent pregnancy loss (RPL) and 60 infertile (inf) PCOS) and 60 healthy non-PCOS controls. Serum levels of hs-CRP were measured by commercial kits, while serum levels of adiponectin and CTRP15 were determined using the ELISA technique. Serum levels of CTRP15 were significantly elevated in PCOS-RPL and PCOS-inf subgroups when compared to controls (94.80 ± 27.08 and 87.77 ± 25.48 vs. 54.78 ± 15.45, both P < 0.001). Moreover, serum adiponectin was considerably lower in the PCOS group and subgroups (P < 0.001), while serum hs-CRP, fasting insulin, HOMA-IR, and free testosterone were significantly higher when compared to the non-PCOS group (P < 0.05). Furthermore, CTRP15 closely associated with FSH, HOMA-IR, hs-CRP, and BMI. These results highlight a possible involvement of CTRP15 in the pathogenesis of PCOS. The elevated levels of CTRP15 might be a compensatory mechanism for the metabolic dysregulations (excess adiposity, insulin resistance, metaflammation) associated with the syndrome. Nevertheless, future studies are necessary to unravel the underlying mechanism.

## Introduction

Polycystic ovary syndrome (PCOS) is one of the most frequent metabolic and endocrine disorders in women affecting 15–20% of women of reproductive age worldwide [1]. In Iran, reports show that 7.1–14.6% of women are diagnosed with the syndrome [2]. PCOS is a complex, heterogeneous disease with an ambiguous etiology, portraying menstrual dysfunction, functional hyperandrogenism, and/or polycystic ovarian morphology [3]. It has been associated with a wide array of endocrinologic dysfunctions [4], chronic low-grade inflammation, glucose and lipid metabolism dysregulation, insulin resistance, and abdominal obesity [5]. Indeed, the interlaced relationship between PCOS and adiposity may further aggravate the risk of infertility and spontaneous abortion in these women [6].

Pregnancy brings forth various metabolic alterations to maintain a positive nutrient supply to the fetus, including reducing insulin sensitivity and reorganizing maternal fat depots. Intriguingly, many pro- and anti-inflammatory cytokines interact to modulate various immunological processes in the uterus necessary for a viable pregnancy; otherwise, abortion, preterm delivery, or preeclampsia may take place [7–9]. Adipose tissue provides energy storage and actively secretes adipokines, such as adiponectin and leptin, which play a vital role in whole-body energy homeostasis [10, 11]. For instance, adiponectin is a pleiotropic hormone that regulates insulin sensitivity, protects against inflammation and atherosclerosis, and modulates reproductive function in both males and females [12]. Leptin regulates food intake and energy balance and is critical in embryo implantation. Both hormones are secreted by endometrial cells [13]. Intriguingly, adiponectin and leptin systems are altered in women suffering from repeated unexplained implantation failure, with reduced expression of leptin, and adiponectin receptors AdipoR1 and AdipoR2 [9, 11, 14]. Recently, Acar et al. reported elevated levels of complement C1q/tumor necrosis factor α-related protein (CTRP)-1 levels in patients with early-onset preeclampsia and late-onset preeclampsia when compared to healthy pregnant women [15].

Adiponectin is a member of a highly conserved family of secreted proteins known as CTRPs (1–15). They share a common structure comprising an N-terminal signal peptide, a short variable region, a collagen repeat domain, and a C-terminal C1q-like globular domain, taking roles in glucose and lipid metabolism, immunity, and inflammation [16, 17]. CTRP15, also known as myonectin and erythroferrone, unlike the other members of the family that are predominantly adipokines, is principally expressed in muscle tissues. The skeletal muscle acts as an endocrine tissue, secreting polypeptide hormones and cytokines–myokines such as Interleukin (IL)-6 and folistatin-like 1, and plays an important role in modulating glucose and lipid metabolism. CTRP15 is a postprandial myokine, released by skeletal muscles in response to the metabolic state. Its expression is suppressed during fasting and diet-induced obesity and is upregulated upon re-feeding [18]. CTRP15 is the liaison between the skeletal muscle and lipid metabolism in the liver and adipose tissue by upregulating CD36, fatty acid-binding proteins, and fatty acid transport proteins, thereby promoting fatty acid uptake [18]. Moreover, it prevents autophagy in the liver by inducing PI3K/Akt/mTOR signaling pathway, indicating a concurrent control and being controlled according to the nutritional status [19]. The hormone is also produced by erythroblasts, modulating iron metabolism and hemoglobin synthesis, and mediating the suppression of hepcidin in stress erythropoiesis [20]. Moreover, Otaka et al. reported that CTRP15 successfully ameliorated apoptosis and suppressed the expression of pro-inflammatory cytokines (TNF-α, IL-6, and MCP-1) in macrophages via sphingosine-1-phosphate/cAMP/Akt-dependent signaling pathway; thereby, protecting against ischemic-reperfusion injures of the heart [21]. However, CTRP15 levels significantly correlated with obesity [22], type II diabetes mellitus (T2DM) [23], gestational diabetes mellitus (GDM) [24], metabolic syndrome (MetS) [25], and coronary artery disease (CAD) [26]. A recent article reported that elevated levels of CTRP15 were observed in CAD patients, and its correlation with insulin resistance and inflammation might be the result of a compensatory mechanism for metabolic dysfunctions in CAD [26].

In regards to the PCOS setting, serum levels of several members of the CTRP family are altered, showing an association with the disease pathogenicity, including CTRP3 [27], CTRP6 [28], CTRP9 [1], CTRP12, and CTRP13 [29]. There is a paucity of research, with only two studies examining the serum levels of CTRP15 in PCOS patients. They reported significantly lower levels of myonectin in PCOS patients when compared to healthy controls, in the Turkish and Chinese populations [2, 30]. Nevertheless, in this study, we will assess the serum levels of CTRP15 in the Iranian population and shed a light on its association with inflammation and metabolic dysregulations in PCOS patients and their fertility status.

## Materials and methods

### Study participants

The study was held under the Declaration of Helsinki and was approved by the Ethics Committee of Kerman University of Medical Sciences, Kerman, Iran (IR.KMU.REC. IR.KMU.REC.1398.616). All participants signed written informed consent. Subjects were selected from the Obstetrics and Gynecology Department of Ibn Sina Infertility Center, Tehran, Iran, and controls from those performing routine checkups at the center, from May 2017 to Jan 2018. Venous blood was collected from each subject at the early follicular phase of their menstrual cycle. The subjects previously participated in our studies, and some of the data have been used in recent publications [28, 31, 32].

The study included 120 subjects diagnosed with PCOS, subdivided into 60 PCOS with Recurrent pregnancy loss (PCOS-RPL) and 60 infertile PCOS (PCOS-Inf) and 60 healthy fertile controls, aged 20–40 years with an average of 29.56 ± 4.14 years (published data) [28, 31, 32]. The inclusion criterion included PCOS diagnosis based on the 2003 Rotterdam Criteria [33], with the exclusion of conditions such as hyperprolactinemia, thyroid diseases, premature ovarian failure, congenital adrenal hyperplasia, Cushing’s syndrome, and adrenal tumors [31]. Subjects allocated to the PCOS-RPL subgroup are defined as those who simultaneously had more than two consecutive abortions earlier than their 20^th^ week of gestation [34]. On the other hand, subjects who were designated to the PCOS-Inf subgroup were those who did not conceive after one year of unprotected sexual intercourse as a result of PCOS pathogenesis. Subjects with other infertility etiologies were excluded. Females allocated to the control group were those who were fertile with regular menstrual cycles and had no clinical/biochemical hyperandrogenism [31]. Women who smoke, are pregnant or lactating, consume alcohol, are diagnosed with diabetes mellitus (DM), cardiovascular diseases (CVD) or hypertension, or had a history of endocrine disorders were excluded from the study. Moreover, participants who have been taking the following medications for the past three months were not included: anti-hypertensive, weight loss agents, anti-inflammatory, lipid-lowering, insulin-sensitizing or euglycemic, antioxidant supplements, and oral contraceptives hyperandrogenism [31].

#### Anthropometrics and biochemical measurements

Anthropometric data and lifestyle factors were recorded for each subject. Body mass index (BMI) was computed as weight (kg) divided by height squared (m^2^). After a 10 h overnight fasting, at the follicular phase of their menstrual cycle, five milliliters of venous blood was collected from each participant as previously described [28]. The serum samples were immediately centrifuged (1000× g for 15 min), aliquoted, and stored at -80°C. Biochemical analyses included fasting blood glucose (FBG), lipid profile (triglycerides (TG), total cholesterol (TC), high density-lipoprotein cholesterol (HDL-C), low density-lipoprotein cholesterol (LDL-C), and high sensitivity C-reactive protein (hs-CRP)), and measured using reliable enzymatic techniques [28, 31, 32]. The serum levels of fasting insulin, follicle-stimulating hormone (FSH), luteinizing hormone (LH), and free testosterone (FT) were measured using ELISA kits as described [28, 31, 32]. The homeostasis model assessment of insulin resistance (HOMA-IR) index was calculated using the following formula: ([FBG (mg/dl)]×[fasting serum insulin (μU/ml)]/405) [35].

#### Measuring adipokines

Circulating levels of adiponectin were measured using the ELISA technique (previously described) [36]. The serum levels of CTRP15 were detected using the ELISA kit (Aviscera Bioscience, Inc., USA; catalog number: SK00393-15). Following the manufacturer’s instructions, we used the intra- and inter-assay coefficients of variants (CV) of 6% and 10%, respectively.

### Statistical analysis

All statistical analyses were carried out using the SPSS 16 software (SPSS, Chicago, IL, USA). First, the Kolmogorov-Smirnov test was used to test the normal distribution of the variables. Normal variables were presented as mean with standard deviation (SD) and compared using Student’s T-test and one-way ANOVA supplemented by Bonferroni post hoc test. Skewed data were presented as median with quartile range, and compared using Mann Whitney U test or Kruskal-Wallis test, with Bonferroni post hoc. Before further analysis, skewed variables were logarithmically transformed to approximate normality. The circulating levels of CTRP15 were also stratified according to BMI as normal weight (BMI ≤ 25 Kg/m^2^) and overweight/obese (BMI > 25 Kg/m^2^), and compared using one-way ANOVA, supplemented with Bonferroni test. Pearson’s correlation coefficients were used to unravel the association of CTRP15 with the other variables. Multiple linear regression analysis was performed using the correlated variables to find out the independent association of CTRP15 with continuous variables. Logistic regression analysis was performed to assess the link between CTRP15 levels and PCOS risk while adjusting for age, BMI, and HOMA-IR. The odds ratio (OR) was defined with a 95% confidence interval (CI). Finally, Receiver operating characteristic (ROC) curve analysis was performed to identify the optimal cut-off level of serum CTRP15 that best predicts PCOS. A p-value of less than 0.05 was considered statistically significant.

## Results

### Characteristics of the study population

The clinical features of the study population are presented in Table 1, a part of which has been reported in our previous studies [28, 31, 32]. There was no significant difference in context of age and BMI between groups (P > 0.05). Regarding the parameters of glucose metabolism, circulating levels of fasting insulin and HOMA-IR were significantly higher in the PCOS group when compared to the non-PCOS group (P < 0.05) (Table 1). Moreover, the lipid profile showed significantly elevated levels of TC and depressed levels of HDL-C in the PCOS group (P < 0.05) than the non-PCOS group. On the other hand, higher levels of FT, LH to FSH ratio, and lower levels of FSH (P < 0.05) were also evident in the PCOS group. The circulating levels of the clinical parameters corresponding to the PCOS subgroups are presented in S1 Table.

**Table 1:** Clinical features of the study population.

Variables	Non-PCOS Group (n = 60)	PCOS Group (n = 120)	*P*-value
**Age** (years)	29.58 ± 4.46	29.64 ± 4.27	0.93^#^
**BMI** (Kg/m^2^)	25.33 ± 3.14	25.92 ± 3.429	0.26^#^
**FBG** (mg/dl)	91.78 ± 9.57	91.13 ± 10.94	0.69^#^
**Insulin** (μU/ml)	2.90 [2.12–4.14]	3.95 [2.67–7.10]	0.001^⁋^
**HOMA-IR**	0.63 [0.45–0.96]	0.81 [0.60–1.54]	0.002^⁋^
**LDL-C** (mg/dl)	95.65 ± 30.61	102.57 ± 29.70	0.14^#^
**HDL-C** (mg/dl)	45.00 [42.00–51.50]	43.00 [39.00–49.00]	0.022^⁋^
**TC** (mg/dl)	161.23 ± 40.58	176.16 ± 36.22	0.01^#^
**TG** (mg/dl)	124.84 ± 35.80	136.14 ± 59.53	0.11^#^
**LH** (IU/L)	6.79 ± 2.65	7.80 ± 4.06	0.08^#^
**FSH** (IU/L)	8.48 ± 2.47	7.39 ± 4.56	0.03^#^
**LH to FSH ratio**	0.87 ± 0.42	1.27 ± 0.78	< 0.001^#^
**FT** (pg/ml)	1.53 ± 0.34	3.13 ± 1.08	< 0.001^#^

Parametric data are given as mean ± standard deviation. Non-parametric data are given as median and interquartile range [25–75%]. P < 0.05 is of statistical significance. PCOS: Polycystic ovary syndrome; BMI: Body mass index; FBG: Fasting blood glucose; LDL-C: Low density-lipoprotein cholesterol; HDL-C: High density-lipoprotein cholesterol; TC: Total cholesterol; TG: Triglyceride; LH: Luteinizing hormone; FSH: Follicle-stimulating hormone; FT: Free testosterone.

# tested by Student t-test

⁋ tested by Mann Whitney u test.

#### Serum levels of CTRP15, adiponectin and hsCRP

Serum levels of CTRP15, adiponectin, and hs-CRP are shown in Table 2. CTRP15 and hs-CRP levels were significantly higher in the PCOS group and subgroups (RPL-PCOS and PCOS-Inf) when compared with the non-PCOS group (P < 0.001). On the contrary, circulating levels of adiponectin in PCOS group and subgroups were significantly lower than that in the non-PCOS group (P < 0.001). Finally, there was no considerable difference in the serum levels of CTRP15, adiponectin, and hs-CRP between the RPL-PCOS and infertile-PCOS subgroups.

**Table 2:** Serum levels of CTRP15, adiponectin and hs-CRP.

Variables	Non-PCOS group (n = 60)	PCOS-Inf subgroup (n = 60)	PCOS-RPL subgroup (n = 60)	*F test*	*P*-value
**CTRP15** (μg/L)	54.78 ± 15.45	87.77 ± 25.48 ^a^**	94.80 ± 27.08 ^b^**	50.7	< 0.001^#^
**Adiponectin** (μg/ml)	5.42 [3.76–8.03]	2.47 [1.55–3.36]^a^**	2.14 [1.95–3.32] ^b^**	-	< 0.001^⁋^
**hs-CRP** (mg/l)	2.46 ± 0.91	4.14 ± 1.21^a^**	3.94 ± 1.30 ^b^**	37.7	< 0.001^#^

Parametric data are given as mean ± standard deviation. Non-parametric data are given as median and interquartile range [25–75%]. *P* < 0.05 is of statistical significance. PCOS: Polycystic ovary syndrome; RPL: Recurrent pregnancy loss; hs-CRP: high-sensitivity C-reactive protein; CTRP15: Complement C1q/tumor necrosis factor-related protein 15.

^⁋^ Kruskal Wallis test

^#^One-way ANOVA with Bonferroni post hoc test

a) Comparison between non-PCOS group and infertile-PCOS subgroup

b) Comparison between non-PCOS group and RPL-PCOS subgroup

* *P* < 0.05

** *P* < 0.01

**Fig 1:**
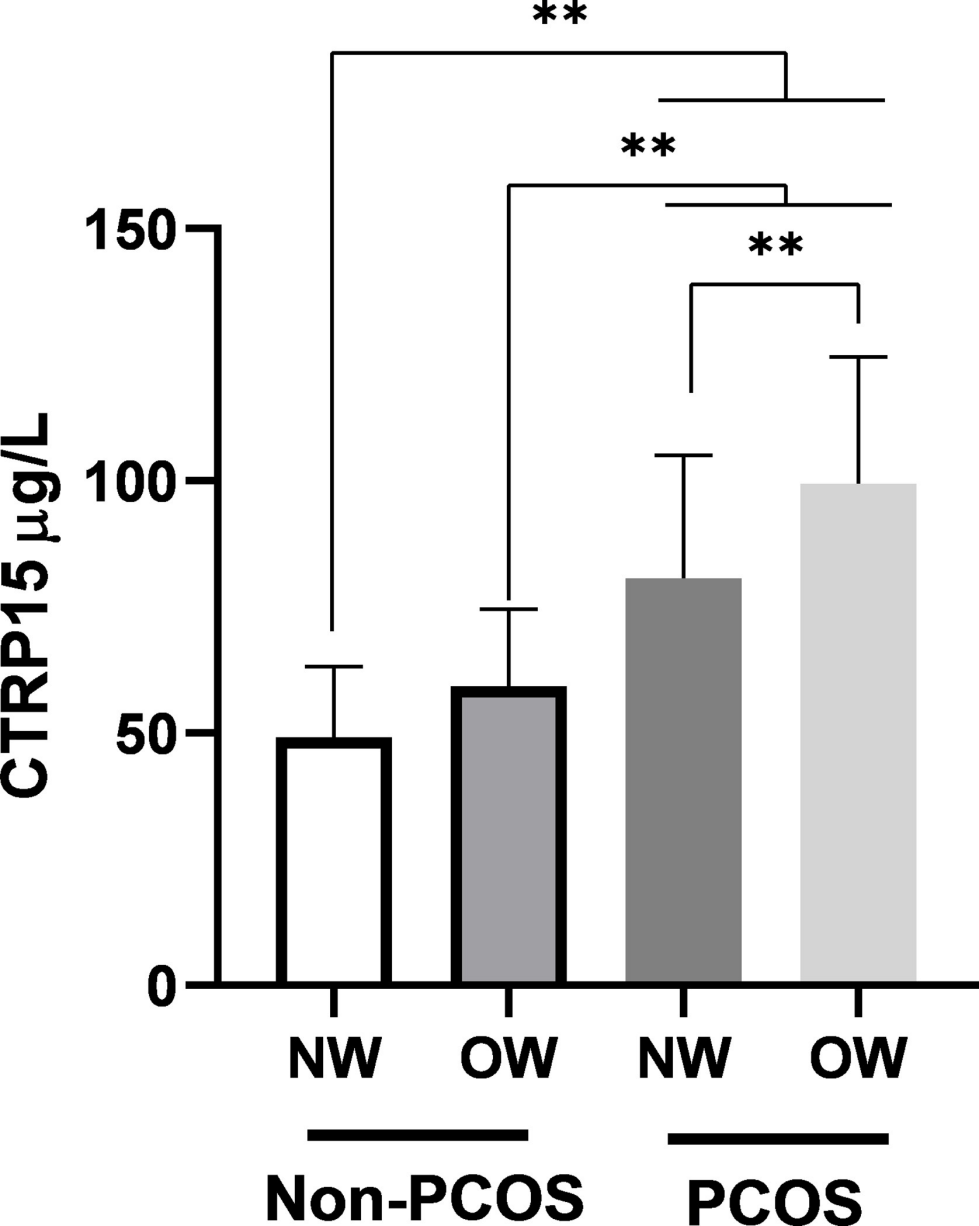
Serum levels of CTRP15 in normal-weight and overweight/obese individuals. Comparison was made using one-way ANOVA supplemented with Bonferroni test. PCOS: Polycystic ovary syndrome; NW: Normal-weight; OW: Overweight/obese. ** P < 0.01.

Next, the circulating levels of CTRP15 were stratified according to BMI as normal-weight (BMI ≤ 25 Kg/m^2^) and overweight/obese (BMI > 25 Kg/m^2^) (Fig 1). Results indicated that CTRP15 levels were significantly higher in normal-weight and overweight/obese PCOS women (89.69 ± 24.42 and 99.40 ± 25.13 ng/ml, respectively) when compared to normal-weight controls (49.18 ± 14.03 ng/ml, P < 0.01). Furthermore, CTRP15 levels were significantly elevated–with the greatest magnitude–in overweight/obese PCOS women as opposed to normal-weight PCOS women and overweight/obese non-PCOS women (59.36 ± 15.25 ng/ml, *P* < 0.01). However, there was no significant difference in circulating levels of CTRP15 between normal-weight and overweight/obese participants in the control group.

### Association of serum CTRP15 with the clinical parameters

Correlations between CTRP15 levels and the measured clinical and biochemical parameters in non-PCOS and PCOS population are shown in Table 3. In the non-PCOS group, circulating levels of CTRP15 significantly correlated with BMI (r = 0.44, P < 0.01) and serum hs-CRP (r = 0.35, P < 0.01). On the other hand, in the PCOS group, it significantly associated with BMI (r = 0.43, P < 0.01), FBG (r = 0.25, P < 0.01), log-insulin (r = 0.24, P < 0.01), log-HOMA-IR (r = 0.29, P < 0.01), TG (r = 0.21, P < 0 .05), FSH (r = 0.19, P < 0.05), and hs-CRP (r = 0.19, P < 0.05).

**Table 3:** Association between CTRP15 with the various clinical variables.

Parameters	CTRP15 (μg/L)					
	Non-PCOS group (n = 60)			PCOS group (n = 120)		
	Pearson Correlation	MLR		Pearson Correlation	MLR	
	R	B (SE)	Beta	R	B (SE)	Beta
**Age**	0.25			0.11		
**BMI**	0.44**	1.9 (0.57) **	0.389	0.43**	2.80 (0.63) **	0.376
**FBG**	0.21			0.25**		
**Insulin**	0.23			0.24**		
**HOMA-IR** ^**a**^	0.24			0.29**	23.04 (7.42) *	0.300
**LDL-C**	- 0.09			- 0.002		
**HDL-C** ^**a**^	- 0.06			0.01		
**TC**	- 0.03			0.08		
**TG**	- 0.02			0.21*	0.037 (0.036)	0.058
**LH**	- 0.098			0.05		
**FSH**	0.03			0.19*	1.08 (0.46) *	0.172
**Free T**	- 0.05			0.01		
**Adiponectin** ^**a**^	- 0.22			- 0.11		
**hs-CRP**	0.35**	4.52 (1.96) *	0.267	0.19*	1.87 (1.73)	0.084

R and R square for MLR in non-PCOS were 0.514 and 0.264, respectively. R and R square for MLR in PCOS were 0.548 and 0.3 and 0.264, respectively.

MLR: Multiple linear regression; B: unstandardized coefficient, SE: standard error. Beta: standardized beta coefficient. Polycystic ovary syndrome; BMI: Body mass index; FBG: Fasting blood glucose; HOMA: Homeostasis model assessment of insulin resistance; LDL-C: Low density lipoprotein-cholesterol; HDL-C: High density lipoprotein-cholesterol; TC: Total cholesterol; TG: Triglycerides; LH: Luteinizing hormone; FSH: Follicle-stimulating hormone; FT: Free testosterone; hs-CRP: high-sensitivity C-reactive protein; CTRP15: Complement C1q/tumor necrosis factor-related protein-15; SE: standard error.

a. logarithmically transformed

* *P* < 0.05

** *P* < 0.01

Next, multiple linear regression (MLR) analyses were performed to assess the independent associations between CTRP15 and metabolic profile (Table 3). Results revealed that serum levels of FSH (β = 82.93, 95% CI [17.86, 14.60]), log-HOMA-IR (β = 34.63, 95% CI [-3.60, 72.87]), hs-CRP (β = 5.02, 95% CI [-3.74, 13.78]), and BMI (β = 34.63, 95% CI [-3.60, 72.87]) independently predicted CTRP15 levels in the PCOS group.

#### Association of serum CTRP15 with the presence of PCOS

Logistic regression analysis was performed to evaluate the independent association between serum levels of CTRP15 and PCOS (Table 4). Results showed a significant association with presence of PCOS (OR 1.086, 95% CI [1.058–1.115], P < 0.001). The association remained significant after adjusting for potential confounders including age, BMI, and HOMA-IR.

**Table 4:** Logistic regression analysis of the effect of the various variable son CTRP15.

Model	β	95% CI		p-value
		Min	Max	
**Crude**	1.086	1.058	1.115	< 0.001
**Model**	1.103	1.1068	1.139	< 0.001

Model: Adjusted for age, BMI and HOMA-IR.

**Fig 2:**
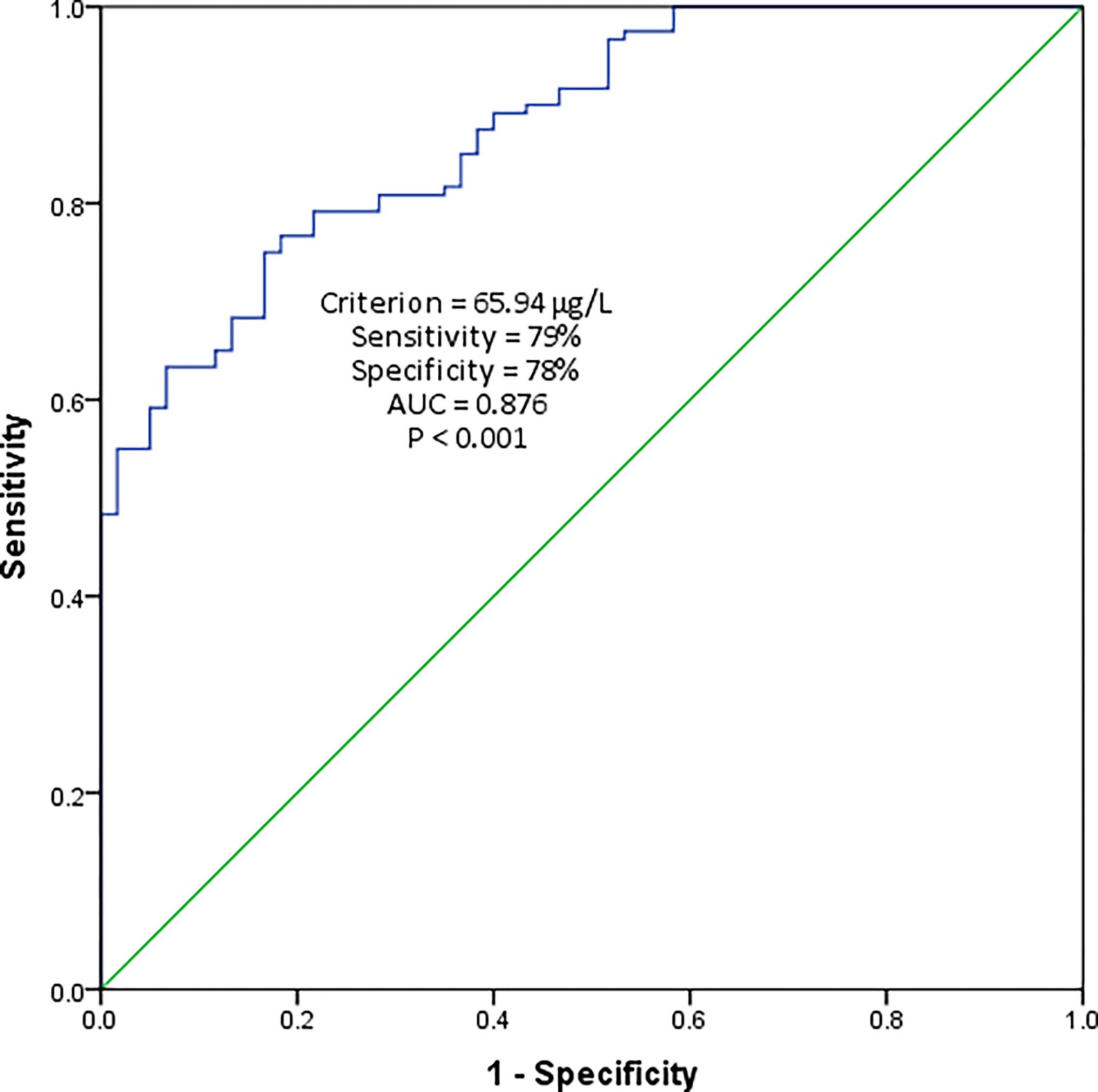
Receiver operating characteristic (ROC) curve analysis for distinguishing of polycystic ovary syndrome (PCOS) using serum CTRP15. AUC: area under the curve.

Finally, ROC curve analysis was operated to validate the ability of serum CTRP15 at predicting PCOS disease (Fig 2). The optimal cut-off point for serum CTRP15 is 65.94 μg/L with area under the curve (AUC) of 0.876 (95% CI [0.827, 0.925], P < 0.001). It was of a relatively good sensitivity of 79% and specificity of 78%.

## Discussion

Adipocyte irregularities with consequential chronic low-grade inflammation and reduced insulin sensitivity are quite common in the pathogenesis of obesity-related metabolic disorders. Of interest, adiposity has been associated with the pathogenesis of PCOS, associating several complications including fertility issues such as RPL. A number of adipokines has been depicted in PCOS pathogenesis including, adiponectin, leptin, Metrnl, and several members of the CTRP family [31, 36, 37]. The present study demonstrated that serum CTRP15 levels were significantly higher in infertile-PCOS and RPL-PCOS women when compared to healthy fertile controls, suggesting that CTRP15 might be involved in the pathogenesis of PCOS.

Previous studies have reported irregularities in the serum levels of other CTRP family members, such as CTRP3 [27, 38], CTRP6 [28, 39] CTRP9 [1, 40], CTRP12, and CTRP13 [29, 41] in metabolic disorders including T2DM and PCOS. CTRP15 is a myokine, mediating the cross-talk between skeletal muscle and other tissues such as adipose tissue and liver to regulate whole-body metabolism [42]. It promotes adenosine monophosphate-activated protein kinase (AMPK) phosphorylation and glucose uptake by glucose transporter type 4 (GLUT4). Furthermore, CTRP15 regulates autophagy via activating AKT kinase/the mammalian target of the rapamycin (mTOR) pathway [25].

CTRP15 has been implicated in a number of metabolic disorders such as MetS, insulin resistance, T2DM, obstructive sleep apnea syndrome (OSAS), and CAD [25, 26, 43, 44]. Mi et al. reported that serum CTRP15 concentration was significantly higher in individuals with MetS and insulin resistance [25]. Furthermore, Li et al. stated that it could be used as a marker in predicting the development of pre-DM and DM [9]. In the pathogenesis of PCOS, only two studies examined the association of CTRP15. Demir and Guler found that CTRP15 levels were significantly lower in Turkish patients diagnosed with PCOS (6.77 ± 1.96 vs. 9.14 ± 2.87 ng/mL, P < 0.001) when compared to healthy controls, predicting PCOS risk [30]. Similarly, Zhang et al. had the same results in the Chinese population (6.51 ± 2.13 vs. 9.35 ± 2.64 ng/mL, P < 0.05) [2]. However, in the present study, we found that the serum myonectin levels are higher in PCOS patients (91.29 ± 26.42 vs. 54.78 ± 15.45 μg/L, P < 0.001) when compared to their healthy counterparts. This discrepancy, along with the elevated serum level, might be due to the different study populations. Inconsistent results on the circulating levels of CTRP15 have been also observed in other metabolic disorders such as T2DM and obesity [23, 45, 46]. Nevertheless, it highlights the possible role of this myokine in PCOS pathogenesis through its association with the metabolic, hormonal, and inflammatory disturbances observed in this syndrome.

Insulin resistance is one of the main pathogenic pillars exacerbating the metabolic disturbances in PCOS. Eighty-five percent of patients diagnosed with PCOS exhibit hyperinsulinemia [5, 47]. CTRP15 is expressed and secreted by skeletal muscle in response to acute nutritional and metabolic changes, as well as a chronic alteration in the energy state of the animals. Glucose and/or fatty acids directly induce CTRP15 expression in myotubes [18]. The molecular mechanism of increasing levels of CTRP15 in patients with PCOS is not clear; however, changes in the regulation of CTRP15 in response to metabolic disorders such as insulin resistance in PCOS individuals could be suggested as a possible mechanism. In the current study, PCOS patients had significantly higher levels of fasting insulin and HOMA-IR when compared to controls (P < 0.05 for both), indicating a degree of insulin resistance. Moreover, there was a direct correlation between serum levels of CTRP15 and the parameters of glucose metabolism (FBG, fasting insulin, and HOMA-IR). Our results come in line with previous studies examining CTRP15 in relation to insulin resistance. For instance, in insulin-resistant subjects without T2DM, CTRP15 levels were significantly higher when compared to the normal controls [48]. Li et al. reported elevated levels of CTRP15 in T2DM subjects when compared to normoglycemic controls, negatively correlating with insulin sensitivity index. The authors suggested that the increased CTRP15 levels may be the result of the impairment of its signaling in target tissues in impaired glucose tolerance (IGT) and T2DM individuals. In addition, they reasoned that the higher levels of CTRP15 might be the consequences of the dysregulation of its synthesis or a response to hyperinsulinemia, hyperglycemia, or cytokines in an insulin-resistant state [9]. Moreover, Shokoohi et al. reported similar results in CAD patients, where the elevated levels of CTRP15 are directly associated with FBG, fasting insulin, and HOMA-IR [26]. Therefore, it can be said that PCOS patients developed CTRP15 resistance; however, future studies are needed to verify this notion. Intriguingly, other studies showed that individuals diagnosed with proliferative diabetic retinopathy or diabetic nephropathy exhibit significantly reduced levels of CTRP15 than those who do not yet have diabetic complications [49, 50]. These conflicting results might be the result of the long-term effect of insulin resistance on skeletal muscle tissue or related to the severity of the metabolic disease [51, 52].

Adiponectin, the high molecular weight (HMW) isoform, in particular, is abundantly produced in subcutaneous fat. The activity of adiponectin is physiologically mediated by enhancing insulin sensitivity through various mechanisms such as induction of AMPK activation resulting in increased cellular nitric oxide (NO) production, fat oxidation, and inhibiting inflammatory effects [53]. Circulating levels of adiponectin are significantly reduced in obesity, T2DM, GDM, and CVD [54–56]. Interestingly, adiponectin regulates fertility and exhibits pro-inflammatory functions in the placenta necessary for the viability of the pregnancy [57]. Numerous studies reported hypoadiponectinemia in females diagnosed with PCOS, correlating with insulin resistance [29, 58]. Senghor et al. also reported lower levels of adiponectin in infertile women with insulin resistance, highlighting that an improvement in insulin resistance increases the chance of fertility [59]. In the current study, we found that adiponectin levels were significantly lower in the PCOS-inf and PCOS-RPL subgroups when compared to the non-PCOS group (P < 0.001). However, we did not find a correlation between CTRP15 and adiponectin levels in PCOS, unlike Shokoohi et al. who found an inverse correlation in CAD patients. Nevertheless, the observed hypoadiponectinemia and a possible myonectin resistance might further exacerbate the ongoing state of insulin resistance in PCOS patients regardless of their fertility status.

Excess adiposity is another factor contributing to the pathogenesis of PCOS. Intriguingly, its relation with serum levels of CTRP15 is quite contradictory. On one hand, an early study by Seldin et al. showed that mice fed on a high-fat diet (HFD) exhibited low serum levels of CTRP15, as a result of reduced mRNA levels in their skeletal muscles, contributing to the reduced free fatty acid uptake and ectopic fat accumulation [42]. This was also seen in other studies [24, 44, 45, 50, 60]. On the other hand, Peterson et al. reported that obese Zucker rats–characterized with a genetic defect in leptin receptor and a constant hyperleptinemia—had elevated levels of CTRP15 [22]. This might be explained by the fact that leptin induces the expression of myonectin in myocytes [61]. Furthermore, serum levels of CTRP15 remained unchanged following a calorie restriction regimen [62]. However, a study by Li et al. reported that CTRP15 levels increased following laparoscopic gastric sleeve in 42 obese patients (P = 0.002), showing an inverse correlation with BMI [60]. In the current study, we found a direct association between BMI and CTRP15 levels. Overweight/obese PCOS patients exhibited significantly elevated levels of the myokine when compared to their normal-weight ones. These findings were contrary to the other two PCOS studies, where they found rather an inverse association between CTRP15 levels and BMI [2, 30]. Again, this difference might be a result of different populations. Nevertheless, the use of BMI in itself is not a gold standard in assessing the fat composition and its relation with physiological and/or pathological factors. It is rather used as ‘a crude estimate’ [63]. Moreover, we found that CTRP15 levels directly correlated with serum TG. This was contrary to what was reported by other studies who rather found that CTRP15 levels were positively correlated with TG and negatively correlated with HDL-C [2, 30]. A recent study by Little et al. reported that CTRP15-deficient mice were characterized by enlarged adipose tissue fat storage, elevated TG levels following HFD, and afflicted lipid clearance following oral lipid load [64]. However, we may propose that excess adiposity leads to a deranged release and/or function of cytokines, culminating in tissue resistance against leptin, insulin, and CTRP15 as well, as a form of a compensatory mechanism.

Moreover, PCOS patients were characterized with disrupted hormonal profile, with significantly reduced FSH and elevated FT (P < 0.05) when compared to healthy controls, with a significant correlation between CTRP15 and FSH levels. Although serum LH levels were higher in the PCOS group, they did not reach statistical significance. PCOS patients do exhibit deranged gonadotropic and androgenic hormones, which are further exacerbated by insulin resistance, resulting in the arrest of follicular growth and subsequent anovulation [47, 65]. Demir and Guler reported an inverse correlation between serum CTRP15 and free androgen index (FAI) in PCOS patients [30]. Moreover, Zhang et al. found that CTRP15 levels negatively correlated with testosterone and positively correlated with sex hormone-binding globulin (SHBG) in the PCOS group [2]. These differences might be the result of the heterogeneity in the study populations. Nevertheless, CTRP15 levels can be associated with the hormonal profile in PCOS patients. PCOS is associated with chronic subclinical inflammation, which is often evaluated by measuring serum levels of hs-CRP [66]. Patients diagnosed with RPL are also characterized by elevated levels of hs-CRP [67]. In the current study, serum levels of hs-CRP were significantly higher in RPL-PCOS and infertile-PCOS subgroups when compared to the non-PCOS group. For the first time, we found a direct correlation between CTRP15 and hs-CRP levels. Consistently, Shokoohi et al. reported a correlation between CTRP15 and inflammatory cytokines (TNF-α and IL-6) [26]. Otaka et al. showed that CTRP15 successfully suppressed inflammatory response in RAW 264.7 macrophages by reducing the expression of TNF-α, IL-6, and MCP-1 [21]. This highlights the role of CTRP15 in the inflammatory process.

ROC curve analysis showed that CTRP15 levels might be a useful marker for PCOS diagnosis. The significantly elevated levels of myonectin in the PCOS group and its correlation with insulin resistance, FSH, TG, and hs-CRP propose that it might be involved in the pathogenesis of PCOS. The elevated levels might be a compensatory mechanism to counteract the effect of insulin resistance, follicular hyperandrogenism, and inflammation. However, future investigations are needed to explore the underlying mechanisms.

However, the present study has some limitations. First, the sample size is relatively small. Second, the results might only apply to the Iranian population and cannot be generalized to other populations. Third, the use of other adiposity parameters such as waist-to-hip ratio, waist circumference, or the use of body composition analyses might have been more beneficial than the mere use of BMI. Fourth, the use of other biochemical parameters of hyperandrogenism such as FAI, SHBG, and anti-müllerian hormone (AMH) might have been more accurate in the assessment of the association between myonectin and hyperandrogenism. Finally, the use of the euglycemic/hyperglycemic clamp is the gold standard for the assessment of insulin resistance; however, HOMA-IR remains widely accepted.

## Conclusion

The current study revealed that PCOS patients with/out RPL exhibit significantly elevated levels of myonectin when compared to controls, contributing to ovarian dysfunction and eventually infertility in these women. Future studies are needed to evaluate the possible use of myonectin as a biomarker in PCOS females suffering from fecundity complications.

## Supporting information

S1 Table Clinical features of the whole population. (DOCX)
