# Supplementary material for: Plasma Complement C1q/tumor necrosis factor-related protein 15 concentration is associated with polycystic ovary syndrome
Source: PLoS One. 2022 Jun 14;17(6):e0263658. doi: 10.1371/journal.pone.0263658 (PMC9197053; doi:10.1371/journal.pone.0263658)
Supplement: S1 Table — (DOCX) [file pone.0263658.s001.docx]

| **Supplementary Table 1 Clinical features of the whole population** | | | | | |
| --- | --- | --- | --- | --- | --- |
| **Variables** | **Non-PCOS Group**  (n = 60) | **PCOS Group**  (n = 120) | **PCOS-Inf subgroup**  (n = 60) | **PCOS-RPL subgroup**  (n = 60) | ***P*-value** |
| **Age** (years) | 29.58 ± 4.46 | 29.64 ± 4.27 | 29.57 ± 4.15 | 29.72 ± 4.43 | 0.93^#^ |
| **BMI** (Kg/m^2^) | 25.33 ± 3.14 | 25.92 ± 3.429 | 25.62 ± 3.47 | 26.23 ± 3.38 | 0.26^#^ |
| **FBG** (mg/dl) | 91.78 ± 9.57 | 91.13 ± 10.94 | 91.73 ± 11.90 | 90.53 ± 9.96 | 0.69^#^ |
| **Insulin** (μU/ml) | 2.90 [2.12, 4.14] | 3.95 [2.67, 7.10] |  |  | 0.001^⁋^ |
| **HOMA-IR** | 0.63 [0.45, 0.96] | 0.81 [0.60, 1.54] |  |  | 0.002^⁋^ |
| **LDL-C** (mg/dl) | 95.65 ± 30.61 | 102.57 ± 29.7 | 102.60 ± 28.46 | 102.55 ± 31.15 | 0.14^#^ |
| **HDL-C** (mg/dl) | 45.00 [42, 51.5] | 43 [39, 49] |  |  | 0.022^⁋^ |
| **TC** (mg/dl) | 161.23 ± 40.58 | 176.16 ± 36.22 | 172.18 ± 35.53 | 180.14 ± 4.75 | 0.01^#^ |
| **TG** (mg/dl) | 124.84 ± 35.80 | 136.14 ± 59.53 | 126.57 ± 58.05 | 145.72 ± 59.94 | 0.11^#^ |
| **LH** (IU/L) | 6.79 ± 2.65 | 7.80 ± 4.06 | 8.80 ± 5.12 | 6.82 ± 2.25 | 0.08^#^ |
| **FSH** (IU/L) | 8.48 ± 2.47 | 7.39 ± 4.56 | 6.25 ± 2.03 | 8.53 ± 5.94 | 0.03^#^ |
| **LH to FSH ratio** | 0.87 ± 0.42 | 1.27 ± 0.78 | 1.45 ± 0.89 | 1.09 ± 0.6 | < 0.001^#^ |
| **FT** (pg/ml) | 1.53 ± 0.34 | 3.13 ± 1.08 | 3.00 ± 0.82 | 3.27 ± 1.29 | < 0.001^#^ |

Parametric data are given as mean ± standard deviation. Non-parametric data are given as median and interquartile range [25, 75%]. Post Hoc analysis are given as mean and median of each group on the far-right column. P < 0.05 is of statistical significance. PCOS: Polycystic ovary syndrome; BMI: Body mass index; FBG: Fasting blood glucose; LDL-C: Low density-lipoprotein cholesterol; HDL-C: High density-lipoprotein cholesterol; TC: Total cholesterol; TG: Triglyceride; LH: Luteinizing hormone; FSH: Follicle-stimulating hormone; FT: Free testosterone. # tested by student t-test, ⁋ tested by Mann Whitney u test.
